# Effect of obesity and low back pain on spinal mobility: a cross sectional study in women

**DOI:** 10.1186/1743-0003-7-3

**Published:** 2010-01-18

**Authors:** Luca Vismara, Francesco Menegoni, Fabio Zaina, Manuela Galli, Stefano Negrini, Paolo Capodaglio

**Affiliations:** 1 Orthopaedic Rehabilitation Unit and Clinical Lab for Gait Analysis and Posture, Ospedale San Giuseppe, Istituto Auxologico Italiano, IRCCS, Via Cadorna 90, I-28824, Piancavallo (VB), Italy; 2 Bioengineering Department, Politecnico di Milano, Italy; 3 ISICO (Italian Scientific Spine Institute), Via Roberto Bellarmino 13/1, 20141 Milan, Italy

## Abstract

**Background:**

obesity is nowadays a pandemic condition. Obese subjects are commonly characterized by musculoskeletal disorders and particularly by non-specific chronic low back pain (cLBP). However, the relationship between obesity and cLBP remains to date unsupported by an objective measurement of the mechanical behaviour of the spine and its morphology in obese subjects. Such analysis may provide a deeper understanding of the relationships between function and the onset of clinical symptoms.

**Purpose:**

to objectively assess the posture and function of the spine during standing, flexion and lateral bending in obese subjects with and without cLBP and to investigate the role of obesity in cLBP.

**Study design:**

Cross-sectional study

**Patient sample:**

thirteen obese subjects, thirteen obese subjects with cLBP, and eleven healthy subjects were enrolled in this study.

**Outcome measures:**

we evaluated the outcome in terms of angles at the initial standing position (START) and at maximum forward flexion (MAX). The range of motion (ROM) between START and MAX was also computed.

**Methods:**

we studied forward flexion and lateral bending of the spine using an optoelectronic system and passive retroreflective markers applied on the trunk. A biomechanical model was developed in order to analyse kinematics and define angles of clinical interest.

**Results:**

obesity was characterized by a generally reduced ROM of the spine, due to a reduced mobility at both pelvic and thoracic level; a static postural adaptation with an increased anterior pelvic tilt. Obesity with cLBP is associated with an increased lumbar lordosis.

In lateral bending, obesity with cLBP is associated with a reduced ROM of the lumbar and thoracic spine, whereas obesity on its own appears to affect only the thoracic curve.

**Conclusions:**

obese individuals with cLBP showed higher degree of spinal impairment when compared to those without cLBP. The observed obesity-related thoracic stiffness may characterize this sub-group of patients, even if prospective studies should be carried out to verify this hypothesis.

## Introduction

Obesity is recognised as a major public health problem in industrialized countries and it is associated with various musculoskeletal disorders, including impairment of the spine [1-3] and osteoarthritis [4,5]. The prevalence of osteoarthritis in obese patients is reported to be 34% (17% at knee, 7% at spine level and 10% other districts), with a significant correlation between body mass index (BMI) and functional joints impairment [6]. The reported prevalence of low back pain (LBP) was 22% on 5724 obese adults 60 years or older, with a linear correlation between LBP and BMI [7].

While body weight is only a weak risk factor for LBP [7], whether obesity is correlated with LBP is still under debate: the association is generally stronger in large population studies than in smaller or occupational studies [7-11]. The BMI-pain association is consistent with what has been observed among persons with obesity seeking weight loss [12,13] and in papers suggesting that weight reduction can reduce reports of musculoskeletal pain [14,15]. Being persistently overweight was associated with disk degeneration at Magnetic Resonance Imaging [16].

When differences in spine biomechanics are investigated, only a moderate link between LBP and BMI appears [3,17-23]. During stance, obese patients show an hyperextension of the lumbar spine [24,25] similar to the anterior translation of the center of mass described by Whitcome in pregnant women [26]. Quantitative evidence exists that excess of weight negatively affects common daily movements, such as standing up [27,28], walking [29-33], lateral bending [34], and forward flexion [35]. Few studies demonstrate a correlation between obesity and functional impairment of the spine secondary to weakness and stiffness of the lumbar muscles, possibly leading to LBP and disability [19,36-38]; moreover, there is a lack of quantitative data on spinal mobility in obese subjects who already suffer from LBP [19].

The aim of our study was to propose a quantitative protocol to describe and quantify the functional mobility of the spine during flexion and lateral bending in order to investigate the relationship between obesity and LBP.

## Materials and methods

Thirty seven adult female volunteers were recruited and divided in three group: 13 obese patients without LBP (Group O) (age: 38.3 ± 8.9 years, BMI: 39.2 ± 3.6 kg/m^2^), 13 obese patients with non-specific chronic LBP [39,40] (Group cLBP) (age: 42.8 ± 11.9 years, BMI: 41.9 ± 5.3 kg/m^2^), and 11 healthy women with no history of musculoskeletal complaints as the control group (Group C) (age: 31.9 ± 8.6 years, BMI: 20.1 ± 1.2 kg/m^2^). We considered three groups of female subjects to take into account the same gynoid mass distribution and because the prevalence of cLBP is greater in women than in men [41]. At the time of the study, cLBP patients were not under any treatment. cLBP patients were defined according to clinical examination and duration of pain [40-42], and all of them performed an X-ray to exclude secondary cLBP. The study has been approved by the local Ethical Committee and all the participants gave written informed consent.

### Experimental setup

The study was conducted at the Laboratory of Gait and Posture Analysis of our Institute. Data were acquired with a 6-camera optoelectronic motion analysis system (Vicon 460, Vicon Motion Systems, Oxford, UK) operating at a sampling rate of 100 Hz. The reflective markers were spherical with diameter of 14 mm.

**Figure 1:**
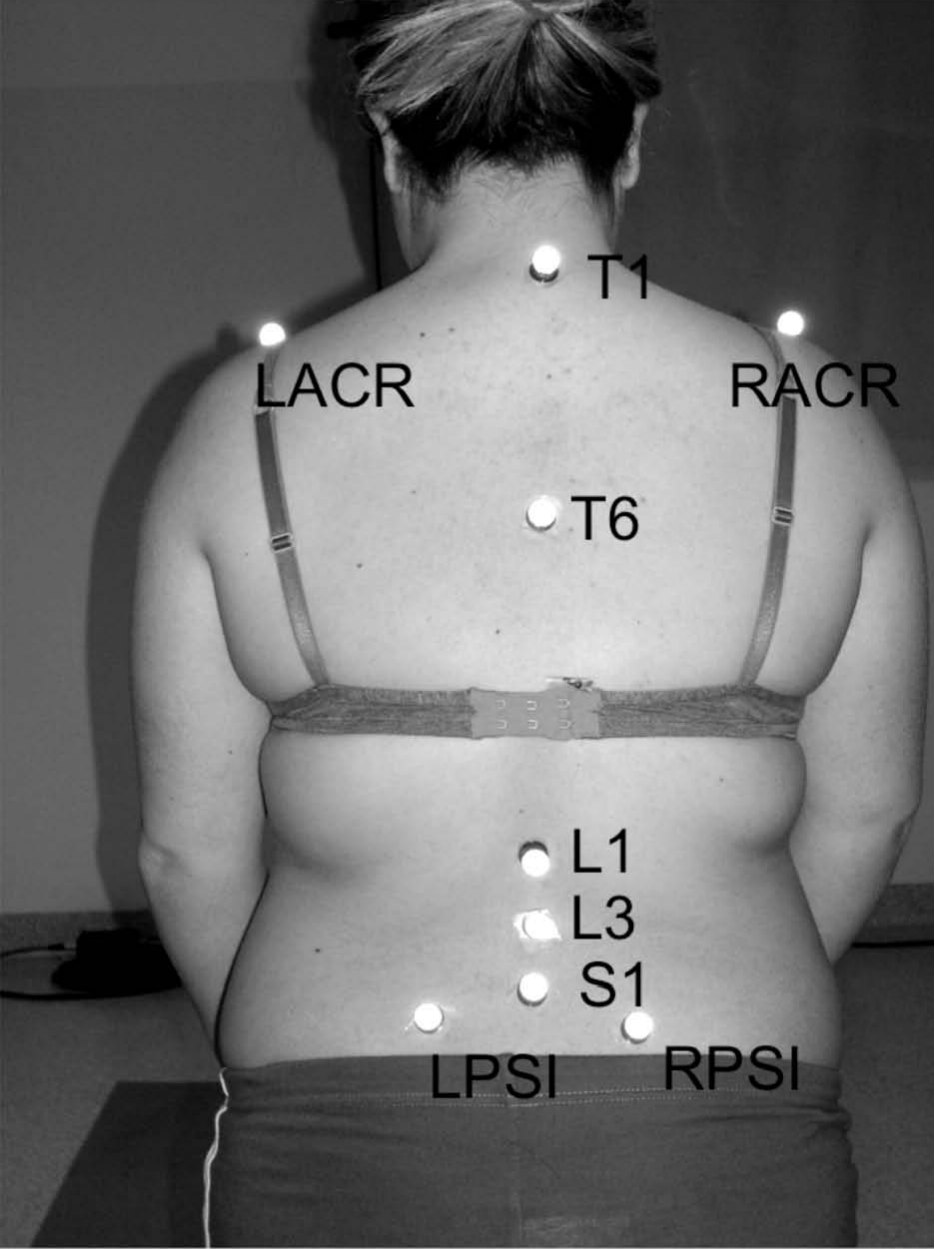
**Marker setup**. Markers were placed on superior posterior iliac spines (LPSI, RPSI), on superior anterior iliac spines (LASI, RASI not visible), on spine spinous processes (S1, L3, L1, T6, T1) and on acromions (LACR, RACR).

The location of the markers, the movements, the angles, and the considered parameters have been previously described [43]. Five markers were placed by the same expert operator along the spine (Figure 1): two on the thoracic (T1 and T6), two on the lumbar vertebrae (L1 and L3), and one on the sacrum (S1). Four markers were positioned on the pelvis: left/right anterior (lASIS/rASIS) and left/right posterior superior iliac spines (lPSIS/rPSIS). Two markers were then applied on the acromion of the left (lSHO) and right shoulder (rSHO).

We analyzed two different tasks: forward flexion and lateral bending both sides. Subjects were instructed to perform the test comfortably at their own preferred speed with feet apart at shoulder width. Each movement was repeated three times and the best acquisition was chosen for further analysis.

### Modelling and data processing

**Figure 2:**
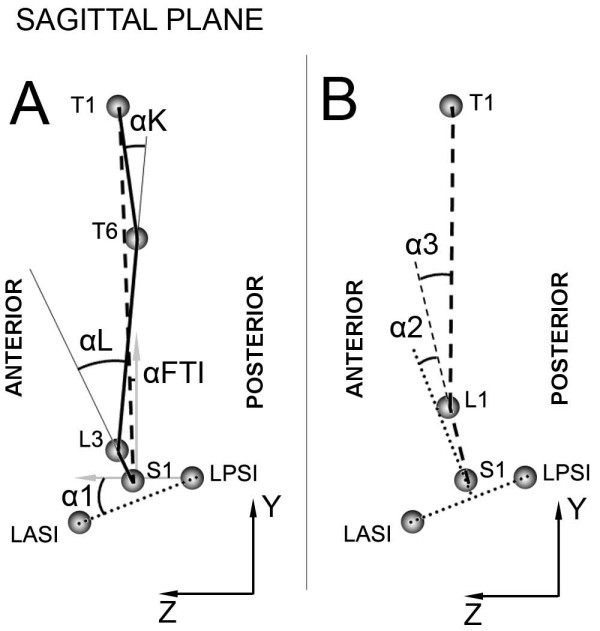
**Representation of markers and angles in sagittal plane during forward flexion**. On the left (Figure 2A) are shown: frontal trunk inclination (αFTI), pelvic obliquity (α1), angle related to kyphosis (αK), angle related to lordosis (αL). On the right (Figure 2B) are represented: lumbar movement (α2), and thoracic movement (α3).

Three-dimensional data from the optoelectronic system were processed using the multi-purpose biomechanical software SMART Analyzer (BTS, Milan, Italy). As for forward flexion, we identified the angles shown in figure 2 to characterize trunk mobility in the sagittal plane, as described in our previous study [43]. We considered: forward trunk inclination (αFTI), anterior pelvic tilt (α1), angle related to lordosis (αL) lumbar movement (α2), angle related to kyphosis (αK), and thoracic movement (α3).

**Figure 3:**
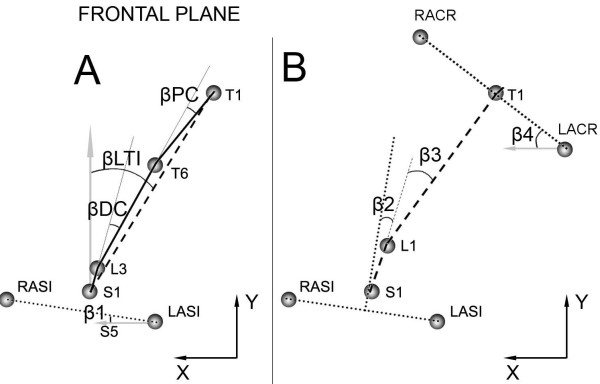
**Representation of markers and angles in frontal plane during lateral bending**. On the left (Figure 3A) are shown: lateral trunk inclination (βLTI), pelvic obliquity (β1), proximal curvature (PC), distal curvature (βDC). On the right (Figure 3B) are represented: lumbar movement (β2), thoracic movement (β3), and angle of shoulders (β4).

The above mentioned angles were evaluated at the initial standing position (START) and at maximum forward flexion (MAX). The range of motion (ROM) between START and MAX was also computed. As for lateral bending, similar angles were considered (Figure 3): lateral trunk inclination (βLTI), pelvic obliquity (β1), lumbar curve (βDC), lumbar movement (β2), thoracic curve (βPC), thoracic movement (β3), and shoulders (β4).

**Figure 4:**
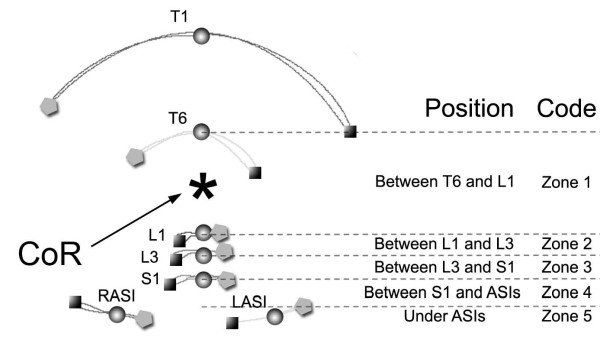
**Lateral bending movement in frontal plane, with representation of markers (sphere: standing position, square: left bending, pentagon: right bending), and the localization of the center of rotation (CoR)**. On the right the code assigned to the CoR to characterize the movement. The represented normal subject was classified as Zone 1, because CoR was located between T6 and L1).

Again the ROM for each angle was evaluated, by computing the difference between maximum left and right bending. We also computed the symmetry index of lateral trunk inclination (βLTI), representing the difference between the maximum left- and right-bend, and the centre of rotation (CoR), a semi-quantitative index used to locate the centre of rotation based on the trajectories of the markers in the frontal plane during the lateral bending. In particular, we identified the CoR by defining different zones delimited by the markers (Figure 4).

### Statistical Analysis

The Statistica software (Statistica 6.0, StatSoft, Tulsa, OK) was used for all the analyses. The Shapiro-Wilk's W test was first used to verify the normal data distribution, and then parametric (one-way ANOVA followed by post-hoc analysis LSD test) or non-parametric (Kruskall-Wallis ANOVA followed by Mann-Whitney U-test with Bonferroni correction) tests were adopted.

## Results

The analyzed groups were not homogeneous in terms of age (ANOVA, p < 0.0001) and BMI (ANOVA, p < 0.0001): specifically, post hoc analysis reported that there were no differences between cLBP and O in terms of age and BMI (p = NS). C was statistically different from the other groups in terms of BMI (post hoc LSD, p < 0.0001). Age was significantly different between C and cLBP (post hoc LSD, p = 0.01).

### Forward Flexion

When compared to C, flexion ROM was reduced in O and cLBP. In the obese subjects, this reduction was mainly influenced by the differences observed during standing posture when compared to C, while for cLBP it was the combination of the reduction in maximum flexion and the standing posture similar to the obese subjects. The angle related to lordosis was significantly increased in cLBP in the start position as compared to C and O. Similar behaviour was observed in MAX but no statistical differences in ROM were evident. The angle related to kyphosis was similar in the three groups in START, but ROM was significantly reduced in O and cLBP.

An increased anterior pelvic tilt angle was present in O and LBP, while no statistically significant reduction in ROM was observed. Lumbar movement in cLBP was significantly reduced in MAX when compared to O as well as to C. In START, statistically significant difference was found only between cLBP and C. The thoracic movement was significantly reduced in O and cLBP as compared to C, not only in MAX but also in ROM (Table 1).

**Table 1:** Main results about the forward flexion movement.

		C	O	cLBP	
		**Mean (SD)**	**Mean (SD)**	**Mean (SD)**	**ANOVA**

*Sagittal Plane*					

Forward trunk inclination (αFTI) [deg]	START (*)	1.2 (2.7)	5.0 (2.5)	4.0 (3.5)	§ p = 0.0093
	MAX (**)	119.4 (9.2)	112.1 (7.5)	103.9 (14.8)	p = 0.0056
	ROM (*,**)	118.2 (9.3)	107.1 (7.5)	99.8 (14.6)	§ p = 0.0041

Anterior pelvic tilt (α1) [deg]	START (*,**)	11.2 (2.4)	20.9 (7.8)	23.9 (8.6)	p = 0.0003
	MAX	72.7 (6.5)	75.2 (13.7)	77.1 (12.4)	NS
	ROM	61.4 (6.2)	54.3 (10.4)	53.2 (9.5)	NS

Angle related to lordosis (αL) [deg]	START (**,***)	30.2 (5.2)	32.7 (8.6)	41.0 (12.9)	p = 0.023
	MAX (*,**,***)	-21.3 (2.6)	-14.6 (5.1)	-5.5 (8.5)	§ p = 0.0001
	ROM	51.5 (5.0)	47.3 (5.9)	46.5 (15.9)	NS

Lumbar movement (α2) [deg]	START (**)	-1.7 (5.1)	-7.8 (13.5)	-15.3 (14.2)	§ p = 0.022
	MAX (**,***)	22.8 (5.2)	19.2 (11.0)	10.9 (11.3)	p = 0.01
	ROM	24.5 (5.6)	27.0 (12.2)	26.1 (12.2)	NS

Angle related to kyphosis (αK) [deg]	START	23.7 (6.4)	25.5 (4.1)	24.9 (5.9)	NS
	MAX (*)	34.6 (8.2)	27.2 (5.5)	29.0 (7.4)	p = 0.048
	ROM (*,**)	10.9 (7.2)	1.8 (5.4)	4.1 (6.4)	p = 0.004

Thoracic movement (α3) [deg]	START	-10.2 (6.7)	-9.0 (14.6)	-4.9 (9.6)	NS
	MAX (*,**)	33.9 (5.2)	25.5 (6.6)	23.4 (9.2)	p = 0.003
	ROM (*,**)	44.1 (8.5)	34.5 (10.0)	28.2 (9.6)	p = 0.001

Trunk, pelvis, lumbar and thoracic values were used in case of forward flexion of the considered segment, negative values otherwise. Negative values of the angle related to lordosis were used to highlight a kyphosis curve of the lordosis segment.

§ Kruskall-Wallis ANOVA,

* differences between C and O (*p *< 0.05)

** differences between C and LBP (*p *< 0.05)

*** differences between O and LBP (*p *< 0.05).

### Lateral bending

cLBP showed a significant reduction in lateral bending and a reduced lumbar ROM as compared to O and C. No differences among groups were observed in lumbar movement and in pelvic obliquity.

The thoracic curve was statistically different among the three groups, with cLBP yielding the worst results. cLBP also showed a significant reduction in thoracic and shoulder movements as compared to O and C (Table 2).

**Table 2:** Main results about the lateral bending movement.

		C	O	cLBP	
*Frontal Plane*		**Mean (SD)**	**Mean (SD)**	**Mean (SD)**	**ANOVA**

Lateral trunk inclination (βLTI) [deg]	START	-0.2 (1.0)	0.7 (1.5)	0.5 (1.7)	§ NS
	ROM (**,***)	77.8 (13.7)	80.7 (8.0)	60.7 (21.3)	p = 0.005

Pelvic obliquity (β1) [deg]	START	-0.5 (1.7)	0.0 (1.6)	-0.2 (2.6)	§ NS
	ROM	12.1 (2.6)	15.2 (4.8)	11.7 (5.6)	§ NS

Lumbar curve (βDC) [deg]	START	1.9 (4.6)	2.1 (3.1)	1.5 (5.5)	NS
	ROM (**,***)	46.0 (7.0)	43.9 (11.3)	29.4 (11.8)	p = 0.0007

Lumbar movement (β2) [deg]	START	-1.9 (1.7)	-0.9 (3.0)	-1.1 (4.2)	§ NS
	ROM	20.1 (8.2)	26.6 (9.3)	21.3 (16.8)	§ NS

Thoracic curve (βPC) [deg]	START	2.2 (2.3)	0.4 (3.1)	0.1 (3.2)	NS
	ROM (*,**,***)	42.2 (9.0)	31.3 (9.0)	23.0 (8.9)	p = 0.00004

Thoracic movement (β3) [deg]	START	2.7 (2.4)	2.8 (2.6)	1.4 (5.3)	NS
	ROM (**,***)	59.2 (9.7)	50.5 (11.8)	35.5 (12.9)	p = 0.00007
Symmetry [deg]		-1.4 (2.5)	0.6 (5.2)	2.5 (6.8)	NS
COR weight	(*,**)	Zone 2	Zone 5	Zone 5	§ p = 0.012

Positive values were used in case of right bending of the segment.

§ Kruskall-Wallis ANOVA,

* differences between C and O (*P *< 0.05)

** differences between C and LBP (*P *< 0.05)

*** differences between O and LBP (*P *< 0.05).

**Figure 5:**
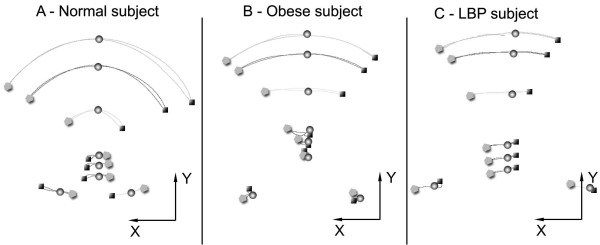
**Lateral bending movement represented in frontal plane (C1, T1, T6, L1, L3, S1, LASI and RASI trajectories) for the different groups**. On the left (Figure 5A) the "hourglass" shape of a normal subject, in the center (Figure 5B) the "cone" shape of a representative obese subject and on the right the "wider cone" shape of a cLBP subject.

The qualitative analysis of lateral bending by locating the CoR showed different trajectories among groups: subjects in C showed an "hourglass" shape (Figure 5A), while O and cLBP showed a "cone" shape (Figure 5B and Figure 5C). CoR was located between L1 and L3 in C (CoR Zone: 2) and between S1 and ASIS in O and cLBP (CoR Zone: 5; Mann-Whitney p = 0.007 and p = 0.012 respectively).

## Discussion

No differences between cLBP and O has been found in terms of age and BMI (p = NS) while, as expected, C was statistically different from other groups in terms of BMI. Age was the only unexpected significant difference between C and cLBP. An age difference may well play a role in obese patients and account for the results obtained by comparisons with controls. However, all the groups were in working age, which is usual in LBP studies, which in turn consider the whole range of working ages.

Our analysis has revealed biomechanical differences in spinal mobility between C and O under static and dynamic conditions. The differences are more pronounced when comparing obese patients with to those without LBP. Prospective studies are needed to prove a cause-effect relationship, but still the gradient of differences observed in the three groups seems to support the hypothesis that obesity modifies spinal posture and function favouring the onset of cLBP. Postural analysis shows significant differences at lumbar and pelvic level among groups. Obesity seems to induce an increase in anterior pelvic tilt while maintaining a normal lumbar lordosis under static conditions. Spinal posture and function and this in turn could favour chronicization of LBP. The increased anterior pelvic tilt induces a greater flexion of the sacroiliac joints, and therefore a higher torque on the L5-S1 joint and discs. This possibly increases the shear forces at this level and overload the disc, thus increasing the risk of disk degeneration [2,16,44]. In line with Gilleard [38], we observed an increased lumbar lordosis in obese patients with cLBP.

Interestingly, women at later stages of pregnancy present the same posture [37]. Obese patients without cLBP, as women at early stages of pregnancy, seem to compensate the forward translation of the center of mass only with an increased anterior pelvic tilt. The increase of lumbar lordosis may well represent a pain-related strategy in obese patients with cLBP.

Abdominal circumference and gravity may influence the lumbar lordosis and its mobility during forward flexion or lateral bending. All these factors could impair the dynamic function of some muscles, in particular the erector spinal muscles, so that their counteraction to the anterior shear forces on the spine could be jeopardized [45]. Postural changes may therefore cause an insufficient muscle force output, but also other factors, such as inappropriate neuromuscular activation and muscular fatigue, may contribute to a reduced spinal stability during full flexion [46].

During forward flexion, we observed that thoracic ROM was significantly lower in O and significantly lower in cLBP as compared to C, while lumbar ROM remained similar among the three groups. Due to thoracic stiffness, forward flexion in O and particularly in cLBP appears to be performed mainly by the lumbar spine, which is most frequently involved in pain syndromes.

Thoracic stiffness with normal lumbar ROM appears to be a feature of obesity and it appears plausible that it might play a role in the onset of cLBP in obese patients.

A rehabilitative spin-off of our study is that targeted exercises for the thoracic spine could prevent the onset of cLBP in obese patients.

In lateral bending, our qualitative analysis based on the location of CoR was able to identify obese (cLBP and O) from their lean counterparts, thus providing a potentially useful clinical index. Further, angular data allowed the identification of obese patients with and without cLBP. In line with McGill [45], our data showed that L3 seems to play a key role in lumbar kinematics.

It has been documented that the lumbar ROM in cLBP can be normal, making questionable its use as an outcome measure. Nevertheless the studies reported by Lehman in his review consider non-obese subjects, and to our knowledge, the lumbar and thoracic ROM have never been studied in obese subjects before [47,48]. Our findings show that obese subjects behave differently to normal weight subjects with and without LBP. In our opinion, this can be considered from a biomechanical point of view as a separate subgroup of cLBP patients that could benefit from a tailored treatment including specific mobilization in addition to the usual rehabilitative approach.

The main limitations of our study include:

➢ The small sample size, due to the time-consuming tests used;

➢ inclusion of females only, to reduce the cross-gender variability of fat mass distribution;

➢ transversal design, to develop hypotheses to be proven in future longitudinal studies;

➢ absence of a not-obese cLBP cohort of patients: including such a group would have allowed to exclude that the results observed were due to cLBP only and not to cLBP and obesity. However, the biomechanical studies on cLBP in not-obese patients showed a higher degree of spinal stiffness, without important postural adjustments such as those observed in our study.

Possibly larger study samples involving non-obese cLBP patient should provide deeper understanding of the relationship between obesity and cLBP and contribute to the identification of different subgroups as the standard deviation values seems to suggest [34].

## Conclusion

Our data show in obese patients static and dynamic adaptations in the kinematics of the spine: under static conditions, obesity *per se *seems correlated to an increased anterior pelvic tilt; under dynamic conditions, to impaired mobility of the thoracic spine. Obesity with cLBP is associated with higher spinal impairment than obesity without cLBP, and an increased lumbar lordosis. Lateral bending is performed in a qualitatively different modality when cLBP is present. It appears the most meaningful clinical test for detecting lower spinal impairments and monitor functional consequences of obesity.

According to our study, even if no cause-effect relationships can be drawn, rehabilitative interventions in obese patients should include strengthening of the lumbar and abdominal muscles as well as mobility exercises for the thoracic spine and pelvis, in line with previous studies [47,49].

The clinical usefulness of an optoelectronic approach is already widely acknowledged in gait analysis for the rehabilitation of several neurological and orthopaedic conditions [50]. Only two studies [43,51] so far has used kinematic analysis of the spine inhealthy subjects. Our study suggests that kinematics of the spine can represent a non-invasive clinically useful technique for functional investigation in various spinal conditions and evaluation of effectiveness in rehabilitation.

## Competing interests

The authors declare that they have no competing interests.

## Authors' contributions

LV designed the study, participated in data collection and analysis, and manuscript writing; FM participated in data analysis, statistical analysis and manuscript writing; FZ participated in the definition of criteria selection of the subject and revision manuscript; MG participated in the study design and the manuscript revised; SN participated to the revision manuscript; PC partecipated to the recruitment of obese patients, study design and gave final approval to the version of the manuscript to be submitted. All the authors approved the final version of the manuscript.
